# Expression of DCX and Transcription Factor Profiling in Photothrombosis-Induced Focal Ischemia in Mice

**DOI:** 10.3389/fncel.2018.00455

**Published:** 2018-11-22

**Authors:** Zhu-Man Lv, Rong-Jian Zhao, Xiao-Song Zhi, Ying Huang, Jia-Yin Chen, Ning-Ning Song, Chang-Jun Su, Yu-Qiang Ding

**Affiliations:** ^1^Department of Basic Medicine, Institute of Neurosciences, Wenzhou Medical University, Wenzhou, China; ^2^Key Laboratory of Arrhythmias, Ministry of Education of China, East Hospital, Department of Anatomy and Neurobiology, Tongji University School of Medicine, Shanghai, China; ^3^Department of Neurology, Tangdu Hospital, Fourth Military Medical University, Xian, China; ^4^Center for Stem Cells and Medicine, Department of Cell Biology, Second Military Medical University, Shanghai, China; ^5^State Key Laboratory of Medical Neurobiology, Institutes of Brain Science, Fudan University, Shanghai, China

**Keywords:** neurogenesis, striatum, ischemia, photothrombosis, Rbp-J

## Abstract

Adult neurogenesis is present in the dentate gyrus and the subventricular zone in mammalian brain under physiological conditions. Recently, adult neurogenesis has also been reported in other brain regions after brain injury. In this study, we established a focal striatal ischemic model in adult mice via photothrombosis (PT) and investigated how focal ischemia elicits neurogenesis in the striatum. We found that astrocytes and microglia increased in early post-ischemic stage, followed by a 1-week late-onset of doublecortin (DCX) expression in the striatum. The number of DCX-positive neurons reached the peak level at day 7, but they were still observed at day 28 post-ischemia. Moreover, Rbp-J (a key effector of Notch signaling) deletion in astrocytes has been reported to promote the neuron regeneration after brain ischemia, and we provided the change of gene expression profile in the striatum of astrocyte-specific Rbp-J knockout (KO) mice glial fibrillary acidic protein (GFAP-Cre^ER^:Rbp-J^fl/fl^), which may help to clarify detailed potential mechanisms for the post-ischemic neurogenesis in the striatum.

## Introduction

Ischemic brain stroke is a serious disease with high incidence and poor prognosis worldwide if systemic thrombolysis or endovascular treatment is not applied in time, and it may result in memory disorder, vascular dementia, affective disorder and ataxia (Donnan et al., 2008; Khandelwal et al., 2016). Now it is well accepted that active adult neurogenesis is present in the mammalian brain although it is largely restricted to the dentate gyrus and the subventricular zone lining the lateral ventricle (Duan et al., 2008; Zhao et al., 2008; Ming and Song, 2011). Recent studies have reported that adult neurogenesis is present the cerebral cortex particularly following cerebral ischemia (Jiang et al., 2001; Pekcec et al., 2006; Kernie and Parent, 2010; Ohira et al., 2010; Huttner et al., 2014). It is challenging and interesting to explore intrinsic mechanism underlying post-ischemic adult neurogenesis, and this line of research may facilitate the development of new therapeutic strategies for restoring brain functions after brain stoke.

The striatum is one major part of basal ganglia with high risk of stroke, and its damage may lead to impairments of voluntary movement and abnormal muscular tension (Fisher, 1965; Nicolai et al., 1996; Feekes and Cassell, 2006). The current methods to build ischemic animal models in adult mammalian brain include the middle cerebral artery occlusion (MCAO; Memezawa et al., 1992), electric coagulation and photothrombosis (PT). However, the infarct area induced by MCAO often covers one third or even larger brain regions in transverse sections, and this large damage is not often present in survived patients. Electric coagulation improves the survival rate with better homogeneity, but the craniotomy changes intracranial pressure, and the surgical approach may damage the temporal vessels, nerves, or muscles (Tamura et al., 1981). PT, firstly reported in 1985 (Watson et al., 1985) can precisely target focal area, and is characterized by easy operation and better controllability for injury location and degree (Fluri et al., 2015). Therefore, PT shows great strengths in building striatal ischemic models (Kuroiwa et al., 2009).

In MCAO-induced cerebral ischemia, reactive astrocytes and nestin-positive neural stem cells could be observed in the striatum, which suggested that striatum has the potential to generate new neurons in extensive ischemia (Shen et al., 2016). However, whether focal ischemia in the striatum can induce neurogenesis is uncertain, although it did elicit active microglial cells and astrocytes locally (Nakajima and Kohsaka, 2004). In our study, we built and verified the striatal ischemic models via PT in adult mice. We recorded changes of reactive astrocytes and microglia and doublecortin (DCX)-positive cells after the striatal ischemia. We also detected the dynamic expression of several transcription factors, which are possibly related to ischemia-induced neurogenesis in our striatal ischemic model. Because the deletion of Rbp-J, a key component of Notch signaling, has been reported to promote neural regeneration in MCAO-induced ischemia (Magnusson et al., 2014), we also investigated the changes of the transcription factors in the striatum of astrocyte-specific Rbp-J knockout (KO) mice in order to provide more insight into this process.

## Materials and Methods

### Animals

C57BL/6J mice were bred in the Laboratory Centre for Medical Sciences, Tongji University. Glial fibrillary acidic protein (GFAP)-Cre^ER^ mice were purchased from The Jackson Laboratory (USA), and floxed Rbp-J mice was generated as described previously (Han et al., 2002; Kuroiwa et al., 2009). GFAP-Cre^ER^:Rbp-J^fl/fl^ mice were obtained by crossing GFAP-Cre^ER^ with Rbp-J^fl/fl^ mice, and litter mates with the genotype of Rbp-J^fl/fl^ were used as control. Male mice aged 8–10 weeks and weighing 18–24 g were used. Before ischemia, the GFAP-Cre^ER^:Rbp-J^fl/fl^ mice were given intragastric administration of tamoxifen (20 mg/ml) once a day for 5 days to induce nuclear translocation of the Cre-ER^T2^ fusion protein and subsequent Cre recombinase activity, allowing deletion of loxP-flanked Rbp-J in GFAP-expressing astrocytes. All animals were bred in-house and maintained in an aseptic environment supplied with water and rodent chow *ad libitum*. All experimental procedures and protocols in the study were approved by the Ethics Committee of Tongji University, China.

### Rose Bengal Photothrombosis Model

**Figure 1:**
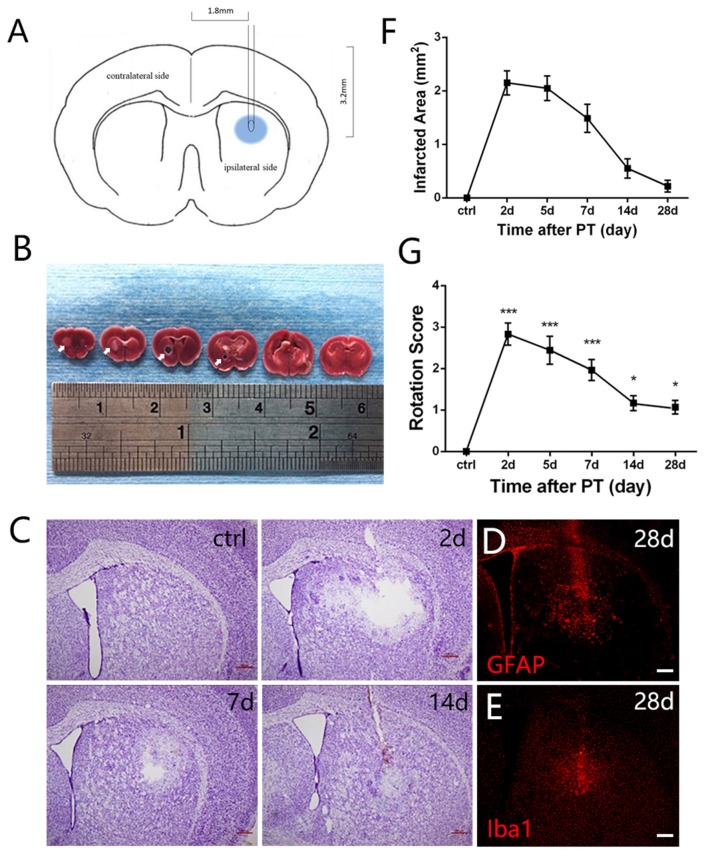
Photothrombosis (PT) induces focal ischemic injury in mouse striatum. **(A)** A diagram shows the experimental paradigm. **(B)** Triphenyltetrazolium chloride (TTC) staining of brain slices. Ischemic areas were indicated by arrows. **(C)** Nissl staining shows a loss of cellular structure in the ischemic region at indicated time points after PT. **(D,E)** Distribution of glial fibrillary acidic protein (GFAP) and ionized calcium binding adapter molecule 1 (Iba1) in the ischemic region at 28 days post PT. **(F)** Quantitative analysis of cellular structure lacking area of (**C**; *n* = 5 in each group). **(G)** The rotation score of mice receiving sham-stroke surgery (control) and mice at indicated time points post PT. Significance was analyzed by compare the mean of 2 days, 5 days, 7 days, 14 days and 28 days with the mean of control (*n* = 5 in each group). Statistical significance: *p < 0.05, ***p < 0.001. Scale bars = 200 μm.

To induce acute striatal ischemic stroke, we applied the rose bengal PT model as described previously (Watson et al., 1985; Yu et al., 2015). A diagram for experimental paradigm is shown in Figure 1A. Mice were under anesthesia with intraperitoneal (i.p.) injection of ketamine-xylazine (65 mg/kg of ketamine, 9.9 mg/kg xylazine) through a round cranial window (2 mm diameter) made at 0.7 mm posterior to the Bregma, and 1.8 mm left to the midline in each mouse. Then a cannula was embedded vertically from the cranial window to the location of striatum 3.2 mm deep, and was fixed by silicate cement. After 1-week recovery from the operation, we injected rose bengal (100 mg/kg mouse) intraperitoneally, and 30 min later, the striatum was illuminated for 10–12 min by inserting an optical fiber with a cold light source (Zeiss FL1500 LCD) from the cannula to induce striatal ischemia. Mice in the control group (sham-stroke, see “Photothrombosis Induces Focal Ischemic Injury in the Striatum” to “DCX Expression Is a Delayed Process After Striatal Ischemia” in “Results” section) received the same operation with inserted optical fiber on one side of striatum and injection of rose bengal, but no illumination was applied.

### Rotational Test

Apomorphine-induced rotation behaviors were examined on mice receiving sham-stroke surgery (control) and mice with PT at 2 days, 5 days, 7 days, 14 days and 28 days. At each time point, apomorphine (10 mg/kg, i.p.; Sigma)-treated mice were placed in Activity Monitor (Med Associates) for 20 min and rotation index was calculated by using EthoVision XT8 software (Noldus) as reported previously (Lerchner et al., 2007; Hu et al., 2014). Rotation scores were calculated by subtracting contralateral from ipsilateral rotations and dividing by the total distance traveled (m), namely rotation scores = rotation numbers/distance (m).

### Quantitative Real-Time PCR (qRT-PCR)

Mice were anesthetized as described above, and brains were removed and cut into 200-μm-thick slices on ice-cold dish to identify the striatum. For detection of ischemia-induced alterations in gene transcripts (see “Changes of Transcription Factors Possibly Involved in the Neurogenesis in Striatum” in “Results” section below), the tissues of striatum of the ischemic side and contralateral intact side were isolated by removing surrounding tissues under dissecting microscopy, and compassion was made between the two sides. For detection of those caused by inactivation of Rbp-J in astrocytes (see “Changes of Transcription Factors in the Ischemic Striatum of Rbp-J KO Mice” in “Results” section below), the tissues of ischemic striatum were isolated in the same way from both GFAP-Cre^ER^:Rbp-J^fl/fl^ mice and control Rbp-J^fl/fl^ mice, and comparison was made between the two genotypes. Total RNA was extracted from each sample but not as a combination of multiple animals’ tissues with TRIzol reagent (Invitrogen, Carlsbad, CA, USA), and 2 μg RNA was reversed to cDNA via SuperScript II reverse transcriptase (Invitrogen) according to the manufacturer’s instructions. All primer sequences were obtained from NCBI database (Supplementary Table S1). qRT-PCR was performed in triplicate for each sample using ABI-7900 (Applied Biosystems, Foster City, CA, USA) with SYBR Green Premix Ex Taq (Takara). The resulting cDNAs were amplified by two-step method under the following conditions: 95°C for 5 min as initial denaturation followed by 40 cycles of denaturation at 95°C for 15 s annealing combined with extension at 60°C for 30 s.

### Triphenyltetrazolium Chloride (TTC) Staining

Mice were anesthetized as described above, and brains were removed immediately and chilled at −20°C for 1 min to slightly harden the tissue. Six 2-mm-thick coronal sections were made from the olfactory bulb to the cerebellum and then stained with 1% triphenyltetrazolium chloride (TTC; Sigma, St. Louis, MO, USA) at 37°C for 30 min.

### Immunohistochemistry and Cell Counting

Mice were harvested at 2 days, 5 days, 7 days, 14 days, 28 days post-ischemia, and mice receiving sham-stroke surgery were used as controls. The mice were anesthetized with an overdose of urethane and then transcardially perfused with 0.1 M PBS followed by 4% paraformaldehyde. Brains were dissected out, post-fixed within 4% paraformaldehyde for 24 h at 4°C, and were sectioned into 30-μm-thick coronary sections on a cryostate. All sections were washed with PBS, blocked with 1% bovine serum albumin (BSA) for 30 min at room temperature, and incubated overnight at 4°C with primary antibodies in PBS containing 0.1% Triton X-100 and 1% BSA. The information about the antibodies and respective dilutions is listed in Supplementary Table S2. After washing in PBS, sections were reacted with the fluorescent-labeled secondary antibody for 3 h at room temperature. Sections were counterstained with Hoechst 33342 (1:2,000; Sigma). Images were obtained with a 50i Nikon fluorescence microscope (Nikon). The numbers of immunostained cells were counted in images taken from ischemic penumbra area (500 μm × 500 μm) using Adobe Photoshop. For non-ischemic mice, number of cells was counted in the equivalent region in the striatum. The counts collected from three to four slices, which covered the whole rostro-caudal extent of ischemic penumbra area with equal distance (30 μm) of each brain were averaged as one value and values from five mice in each group were averaged as a group value.

### BrdU Incorporation Assay

To label newly synthesized DNA, animals received i.p. injection of BrdU (50 mg/kg; Sigma) four times, 48 h, 36 h, 24 h and 12 h before harvest. For BrdU staining, brain sections were heated at 37°C for 30 min in 2 M HCl, then neutralized with 0.1 M borate buffer (pH 8.5) for 10 min and incubated overnight with a rat monoclonal antibody against BrdU (1:1,000; AbD Serotec) followed by a rhodamine-conjugated goat anti-rat secondary antibody (1:500; ZSGB-BIO) for 2 h at room temperature. The nucleus was counterstained with Hoechst 33342 (Sigma).

### Nissl Staining

Thrity micrometer-thick sections mounted on gelatin-coated slides were incubated in toluidine blue pH 4.1 for 30 min, dehydrated through a battery of alcohols with increasing graduation, xylene transplant and coverslipped in neutral balata.

### Statistical Analysis

**Figure 2:**
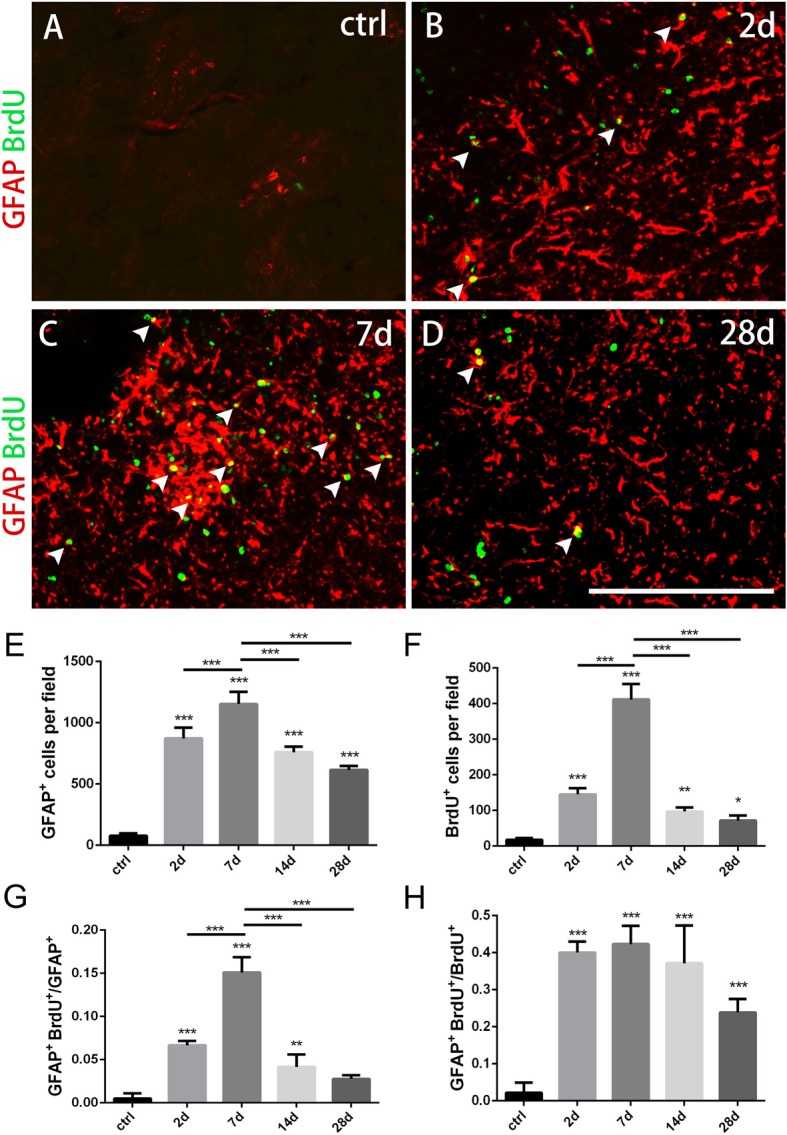
Changes of astrocytes in the striatum after PT-induced ischemia in mice. **(A–D)** GFAP^+^ astrocytes and BrdU-incorporated proliferating cells in uninjured mice (ctrl) and post-ischemic mice during the time course (2 days, 7 days, 14 days and 28 days, respectively). **(E)** Quantitative analysis of GFAP^+^ astrocytes at indicated time points after ischemia. **(F)** Quantitative analysis of BrdU-incorporated proliferating cells at different time points after ischemia. **(G)** Ratio of GFAP/BrdU-double labeled cells to the total of GFAP^+^ cells at different time points after ischemia. **(H)** GFAP/BrdU-double cells to the total of BrdU^+^ cells at different time points after ischemia. Arrowhead marks the GFAP/BrdU-double cells. **(E–H)** *n* = 5 in each group. Statistical significance: *p < 0.05, **p < 0.01, ***p < 0.001. Scale bars = 200 μm.

**Figure 3:**
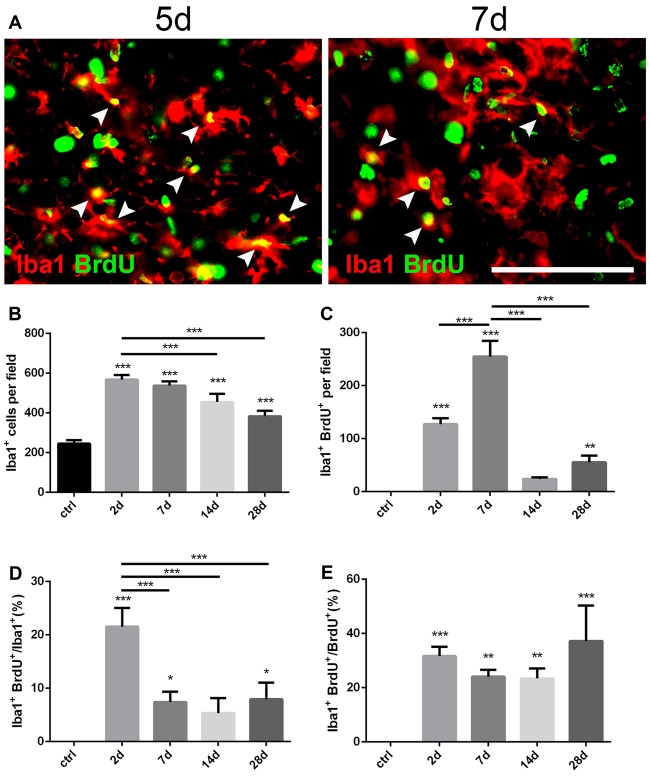
Changes of microglia in the striatum after PT-induced ischemia in mice. **(A)** Iba1^+^ microglia and BrdU-incorporated proliferating cells in post-ischemic mice at day 5 and day 7. Arrow heads point to Iba1/BrdU-double labeled cells. **(B)** Quantitative analysis of Iba1^+^ microglia at indicated time points after ischemia. **(C)** Quantitative analysis of Iba1/BrdU-double labeled cells at indicated time points after ischemia. **(D)** Ratio of Iba1/BrdU-double labeled cells to the total of Iba1^+^ cells at indicated time points after ischemia. **(E)** Iba1/BrdU-double labeled cells to the total of BrdU^+^ cells at indicated time points after ischemia. **(B–E)** *n* = 5 in each group. Statistical significance: *p < 0.05, **p < 0.01, ***p < 0.001. Scale bars = 100 μm.

**Figure 4:**
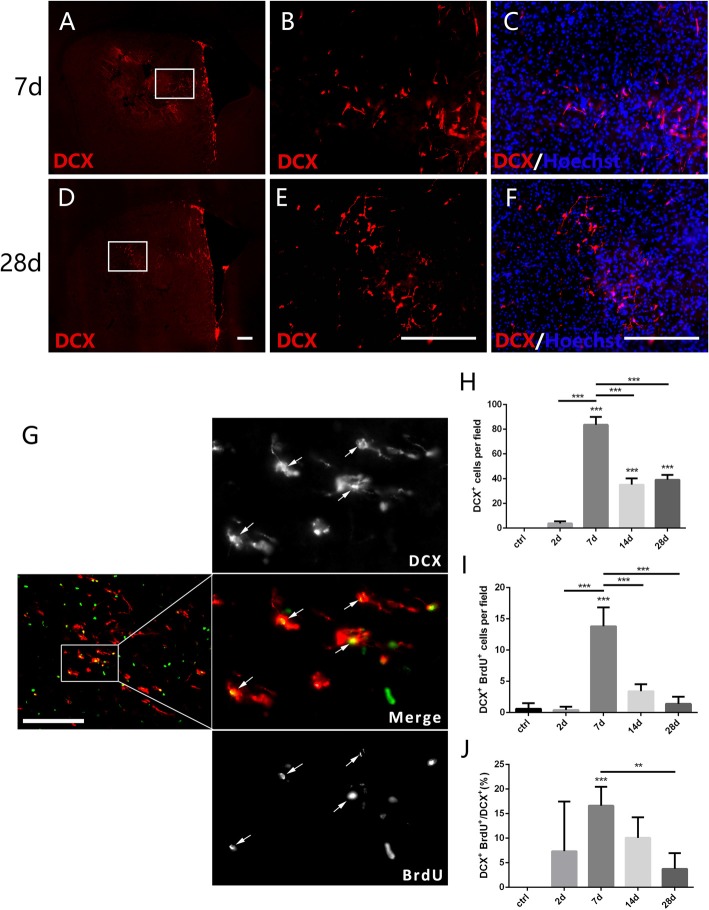
New-born neurons in the striatum after PT-induced ischemia in mice. Panels **(A–F)** showing doublecortin (DCX^+^) cells in the striatum at 7 days **(C)** and 28 days **(F)** post-ischemia. Boxed areas in **(A,D)** are enlarged in **(B,E)**, respectively. Panel **(G)** showing DCX/BrdU-double positive cells (arrows) at day 7 post-ischemia. **(H–J)** Quantitative analysis of DCX^+^ cells, DCX/BrdU-double positive cells and ratio of DCX/BrdU-double positive cells to the total of DCX^+^ cells at indicated time points post-ischemia, respectively. **(H–J)** *n* = 5 in each group. Statistical significance: **p < 0.01, ***p < 0.001. Scale bars = 200 μm.

**Figure 5:**
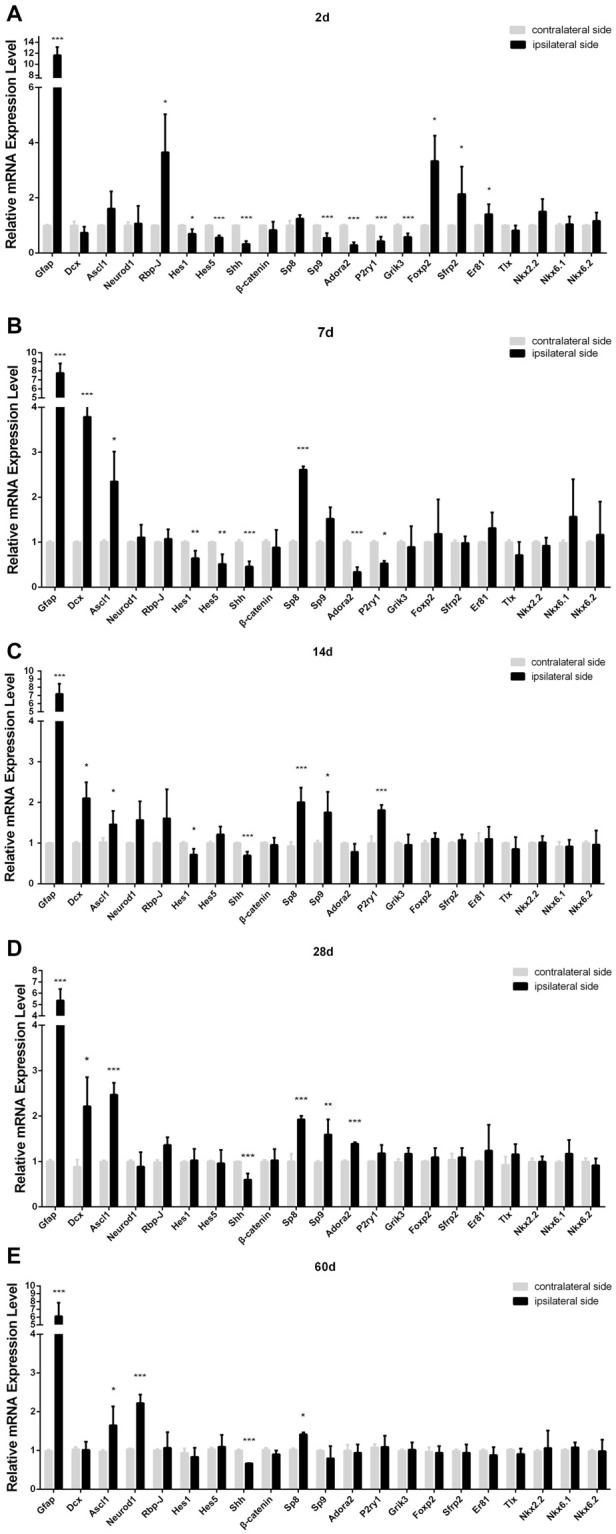
Relative mRNA level of the genes in ischemic striatum relative to contralateral striatum at day 2 **(A)**, 7 **(B)**, 14 **(C)**, 28 **(D)**, 60 **(E)** post-ischemia. Ipsilateral side: stroke side; contralateral side: uninjured side. *n* = 4 in each group. Statistical significance: *p < 0.05, **p < 0.01, ***p < 0.001.

**Figure 6:**
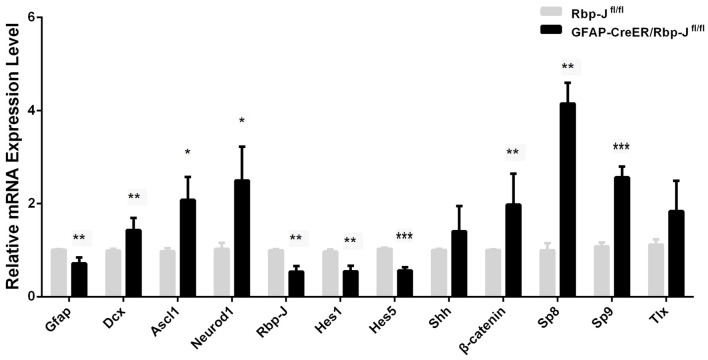
Relative mRNA level of the genes in the striatum of GFAP-Cre^ER^/Rbp-J^fl/fl^ mice (*n* = 4) to Rbp-J^fl/fl^ (*n* = 4) at 7 days post-ischemia. Statistical significance. *p < 0.05, **p < 0.01, ***p < 0.001.

All experiments were performed for at least three biological repeats. Data was reported as mean ± SD. Statistical analysis and diagrams were performed with GraphPad Prism 6.0. Significance of results was analyzed using one-way ANOVA (more than two groups were compared, i.e., Figures 1–4) followed by Tukey’s test procedure for multiple comparisons with false discovery rate (FDR) correction, or two-tailed Student’s *t*-test (two groups compared, i.e., Figures 5, 6). *P* < 0.05 was considered statistically significant.

## Results

### Photothrombosis Induces Focal Ischemic Injury in the Striatum

Mortality rate of mice treated with PT was low in our experiments (2.5%). To test whether striatal ischemic injury model was built successfully by PT in mice, we performed TTC staining, Nissl staining and apomorphine-induced rotational test. Macroscopically, TTC staining clearly showed the white-stained ischemic area in the ipsilateral striatum 24 h post PT (arrows, Figure 1B). In contrast, the contralateral brain was red-stained (Figure 1B). Nissl staining showed an obvious loss of cellular structure in the ischemic region at day 2, and 5, but it became smaller at day 7 and 14 and was repopulated with Nissl-stained cells at day 28 after the focal ischemia, most of which were GFAP-positive astrocytes and ionized calcium binding adapter molecule 1 (Iba1)-positive microglia (Figures 1C,E). Loss of cellular structures in ischemic core is likely caused by the procedures of Nissl staining, as this situation was also observed when PT was performed in the cerebral cortex with intact skull.

To evaluate the locomotive deficit induced by the striatal injury, we performed apomorphine-induced rotational test, which is widely used to examine the dopamine-involved striatal activity (Arenkiel et al., 2008). The rotation score is calculated by subtracting contralateral from ipsilateral rotations and dividing by the total distance traveled. The score can be used to reflect neuronal activity in the striatum (Hu et al., 2014) and may be helpful for evaluation of functional recovery in future studies. It was nearly zero before the ischemia. At day 2 post-ischemia, the score reached the peak value (nearly 3), maintained high level at day 5 and day 7, and dropped down significantly at day 14 and day 28 but still higher than the base level (Figure 1G). These results indicated that locomotive deficit was induced by the focal PT in the striatum.

### Astrocytes Are Activated After the Striatal Ischemia

Astrocytes play a key role in the maintenance of normal brain functions as well as post-injury restoration. Upon injury, quiescent astrocytes transformed into reactive astrocytes with strong proliferation ability and high GFAP expression, and ended in glial scar in damage area (Lu et al., 2014). More importantly, astrocytes were regarded as potential cell source for neurogenesis (Guo et al., 2014; Lu et al., 2014; Magnusson et al., 2014).

To test whether astrocytes were activated in our ischemic model as well as their proliferation ability, we performed immunostaining of GFAP combined with BrdU that was applied four times before the mice were sacrificed. GFAP^+^ astrocytes were scattered in intact striatum. However, GFAP^+^ cells dramatically increased at day 2, reached the peak level at day 7, and gradually dropped down to day 28 post-ischemia (Figures 2A–E). Similarly, BrdU^+^ cells were rare in intact striatum, but it increased significantly at day 2, reached the highest level at day 7, and reduced at day 14 and day 28 (Figures 2A–D,F). Furthermore, we calculated the ratio of GFAP^+^/BrdU^+^ cells to GFAP^+^ cells and ratio of GFAP^+^/BrdU^+^ cells to BrdU^+^ cells, and found that both of them reached the highest level at day 7 (15.1 ± 0.69% and 42.09 ± 2.56%) (Figures 2A–D,G,H). These results demonstrated that the astrocytes were highly proliferated in our striatal ischemic model.

Morphologically, GFAP^+^ astrocytes changed its shape significantly in the ischemic region over time after PT. In the early period (2 days, 7 days), GFAP^+^ astrocytes exhibited a typical stellate-like morphology with extensive processes. However, astrocytes were densely packed and formed a stream with elongated, straight shape in the ischemic core at late stage, especially at day 28 (Figure 1D).

### Microglia Are Activated After the Striatal Ischemia

Iba1 is a marker for quiescent as well as activated microglia (Zamanian et al., 2012). To investigate the dynamic changes of microglia after PT, we performed double immunostaining of Iba1 and BrdU (Figure 3A). Iba1^+^ cells were highly proliferated in the ischemic area from the initial 2 days after PT, and then dropped down over time but still maintained a high level even at day 28 (Figures 1E, 3B). The peak level of Iba1^+^/BrdU^+^ cells was observed at day 7 (254.9 ± 17.15; Figure 3C), and the highest ratio of Iba1^+^/BrdU^+^ cells to Iba1^+^ cells was present at day 2 (21.55 ± 2.03%; Figure 3D), suggesting that microglia owed the fastest proliferation rate within the first week after striatal ischemia. On the other hand, the ration of Iba1^+^/BrdU^+^ cells to BrdU^+^ cells at day 2 and day 7 were 31.67 ± 1.95% and 24.07 ± 1.49% (Figure 3E), respectively. Overall, Iba1^+^ microglia were highly and quickly activated after the striatal ischemia.

### DCX Expression Is a Delayed Process After Striatal Ischemia

DCX is a microtubule associated protein in cytoplasm, and can be used to mark the new-born neurons (des Portes et al., 1998). To test whether the focal ischemia is able to trigger appearance of new-born neurons in the injured striatum, we examined DCX-expressing cells. DCX^+^ cells were not detected in the striatum at day 2 when astrocytes were quickly expanded, but were observed at day 7 and continuously existed at day 28 post ischemic injury (Figures 4A–F,H). Moreover, the number of DCX^+^/BrdU^+^ cells and the ratio of DCX^+^/BrdU^+^ cells to DCX^+^ cells both reached the highest level at day 7 (Figures 4G,I,J). In comparison with the quick reaction of astrocytes and microglia, the ischemia-evoked neurogenesis is a delayed process in the striatum.

### Changes of Transcription Factors Possibly Involved in the Neurogenesis in Striatum

Our PT-induced ischemia did activate the astrocytes and microglia, which was companied by a delayed occurrence of DCX^+^ cells in the striatum. In order to uncover the key genetic regulators controlling this process, we used qPCR to compare the expression of a certain number of transcription factors and signaling pathways that have been reported to be involved in the morphogenesis of striatum between the ipsilateral side (ischemic side) and the contralateral side (uninjured side), on the basis of the hypothesis that these genetic programs may be reinitiated during the ischemia-induced striatal neurogenesis. Among them, Notch signaling pathway is one of the most important networks, because the deletion of *Rbp-J*, a canonical pathway effector for Notch signaling pathway, has been reported to promote neural regeneration in MCAO-induced ischemia (Bhat, 2014; Magnusson et al., 2014). We found that *Rbp-J* was transiently up-regulated at day 2 and then returned to the normal level (Figure 5). However, *Hes1* and *Hes5*, target transcription factors of Notch signaling pathway, were down-regulated during the early post-ischemic process, then returning to normal state at late stage (Figure 5).

Transcription factor Sp9 is known to promote striatopallidal medium-sized spiny neuron progenitor division and differentiation (Zhang et al., 2016). Our results showed that the mRNA of *Sp8*, a member of Sp family, significantly increased at day 7, three times higher than that in the contralateral side, and still maintained high level during later period (Figure 5). However, *Sp9* was slightly down-regulated at day 2, but its expression was all higher than that in the contralateral side at day 14 and day 28. *Adora2*, *P2ry1*, *Grik3*, the target factors of *Sp9* (Zhang et al., 2016), showed the similar changes to that of *Sp9* (Figure 5).

*Ascl1*, also called* Mash1* and *Neurod1* are neurogenic genes, and importantly, a single overexpression of *Ascl1* or *Neurod1* in astrocytes could make trans-differentiation of astrocytes into functional neurons (Guo et al., 2014; Liu et al., 2015; Brulet et al., 2017). In our study, the trend of *Ascl1* expression change was similar to *Sp8*, no statistically different at day 2 but higher than the contralateral side at day 7, 14, 28 and 60 post-ischemia. *Neurod1* also showed higher expression at the late stage post-ischemia (day 14, 28 and 60; Figure 5).

Shh signaling is involved in multiple organogenesis including the nervous system, and its expression is required for the proliferation, differentiation and migration of neural precursors and neurogenesis in the cortex (Jin et al., 2015). However, in our PT-induced ischemic model, *Shh* was down-regulated at both early and late stages (Figure 4). Besides, *β-catenin*, a key component of Wnt singling pathway (Nusse and Clevers, 2017), was unchanged in our ischemic model (Figure 5).

### Changes of Transcription Factors in the Ischemic Striatum of Rbp-J KO Mice

Magnusson et al. (2014) found that Notch signaling was up-regulated, and conditional KO of *Rbp-J* in the astrocytes could promote the trans-differentiation of astrocytes into neurons in MACO-induced ischemia. In this study, we created GFAP-Cre^ER^:Rbp-J^fl/fl^ mice, in which Rbp-J was inactivated specifically in GFAP-expressing astrocytes in the presence of tamoxifen. We induced the striatal ischemia in both GFAP-Cre^ER^:Rbp-J^fl/fl^ mice and Rbp-J^fl/fl^ (control). At day 7 post-ischemia, when DCX^+^ neurons were first observed after the injury, we focused on comparison of transcriptions of the genes in the striatum of both GFAP-Cre^ER^:Rbp-J^fl/fl^ mice and the Rbp-J^fl/fl^. We found that *Hes1* and *Hes5*, target factors of Rbp-J (Kageyama et al., 2008), were both reduced in GFAP-Cre^ER^:Rbp-J^fl/fl^ mice relative to control Rbp-J^fl/fl^ mice (Figure 6). Interestingly, the expression of *GFAP* was also down-regulated, and this might suggest less appearance of astrocytes in GFAP-Cre^ER^:Rbp-J^fl/fl^ mice. In contrast, the expression of *DCX* was statistically higher in GFAP-Cre^ER^:Rbp-J^fl/fl^ mice than that in control Rbp-J^fl/fl^ mice. Besides, the levels of *Ascl1*, *Neurod1*, *β-catenin*, *Sp8* and* Sp9* were also higher than the control group (Figure 6). These results suggested that Rbp-J deletion may promote neurogenesis via multiple neurogenic genes.

## Discussion

PT is easy-operated, and importantly its target site is easy to control. PT-induced focal ischemia in mouse striatum could well mimic human striatal ischemia, which may help to reveal the mechanism of neurogenesis caused by ischemia and find potential therapeutical target.

In this study, mice receiving the PT showed obvious locomotive deficits, suggesting that the mice did suffer striatal damage. In many cerebral diseases, the injured area was always accumulated by reactive glial cells, including astrocytes and microglia. This process was recognized to form glial scar, so as to avoid infection, repair injury area and reconstruct the integrity of neuronal connections (Liddelow and Barres, 2016). Recent studies have shown that reactive astrocytes could be one main cell source of neurogenesis during recovery (Magnusson et al., 2014). In our work, large amounts of astrocytes appeared at day 2 and reached the highest level at day 7 post-ischemia. Later on, GFAP^+^ astrocytes were densely packed with broad processes (i.e., astroglial scar formed). Astroglial scar is recognized as protective reaction, but may also result in refractory epilepsy. If reactive astrocytes can be transformed into neurons under ischemic condition, it is of profound significance to find the fittest time and proper treatment on the astrocytes not only in aid of neural regeneration but also to avoid astroglial scar-induced side effects.

Previous studies have shown that cerebral neurogenesis can be triggered by two programs: (i) adult neural stem cells, located in the subventricular zone and hippocampal subgranular zone, continuously produce new neurons during whole lifetime (Braun and Jessberger, 2014); when activated by external signaling, they migrate to certain areas (e.g., ischemic area) and form lineage-restricted neurons or astrocytes to reconstruct functional neural unit (Yagita et al., 2001); and (ii) some specialized astrocytes, possessing similar properties to neural stem cells and an intrinsic ability to generate neurons. Although this ability is dormant, it can be brought out by stimulating the cells with growth factors *in vitro* (Sirko et al., 2013). Recently, it has been reported that some parenchymal astrocytes in the striatum can also produce neurons *in vivo* (Magnusson et al., 2014; Nato et al., 2015). Ischemia was reported to have intense inductive effect on neurogenesis (Yagita et al., 2001; Magnusson et al., 2014). In our study, we found that focal striatal ischemia could also elicit the occurrence of DCX^+^ cells in adult mice, although it was a delayed process compared to glial cells. Further studies are needed to identify the origin of the DCX^+^ cells, generated locally or adult neural stem cells migrated from the wall of lateral ventricle.

The cell homoeostasis of certain state is maintained by stabilized intrinsic gene expression, and the disturbance of cell signaling network could trigger the change of the cell state. In our study, PT-induced ischemia would break the homoeostasis, activating the astrocytes and promoting the neurogenic program of DCX^+^ cells, which may be paralleled by complicated changes in transcriptional network. In order to uncover the potential mechanism within the recovery of post-ischemic injury, we detected the dynamic expression of several transcription factors that is implicated in the striatum development in the view of similar signaling network between them. We found that the key effector of Notch signaling, *Rbp-J*, was transiently up-regulated and then returned to normal level. Factors which are indispensable for the striatal neurogenesis (e.g., *Ascl1*, *Neurod1* and *Sp* family) were up-regulated during mid and late period. These results suggest that the amplification of astrocytes in the early phase might be mediated by Notch signaling activation, and it becomes inhibited when neurogenic program is launched.

Reactive astrocytes usually exist in the injury area and express stem properties of neural stem cells, likely to be a potential cell source for *in vivo* reprogramming. It has been reported that some transcription factors (e.g., *Neurod1*, *Ascl1*, *Pax6* and *Dlx2*) could reprogram astrocytes into neural progenitors or functional neurons *in vivo* (Heinrich et al., 2011; Jang and Goldman, 2011; Guo et al., 2014; Liu et al., 2015). Besides, *in vivo* study showed that overexpression of single factor Sox2 was sufficient for trans-differentiation from astrocytes to neural precursors, and the neural state could maintain through whole lifetime (Niu et al., 2013). The striatum contains several specified types of functional neurons, and the genetic program governing ischemia-induced transformation of astrocyte into neurons should have something unique in addition to the pan-reprogram machinery. In this study, we identified the changes of genes that are implicated in the development of striatal neurons under ischemic condition, and they may be candidate genes for exploring the mechanisms underlying striatal ischemia-induced neurogenesis.

It has been reported that Rbp-J deletion in astrocytes enhances their proliferation ability spontaneously after 2–3 weeks, and promotes the generation of Ascl1^+^ and DCX^+^ cells in MCAO-induced ischemia (Magnusson et al., 2014; Magnusson and Frisén, 2016). Consistently, our study showed that the level of *DCX* transcripts was statistically higher in GFAP-Cre^ER^:Rbp-J^fl/fl^ mice than that in control Rbp-J^fl/fl^ mice after the striatal ischemia. In addition, the levels of neurogenic genes (e.g., *Ascl1* and *Neurod1*) and transcription factors controlled the striatal neurogenesis (e.g., *Sp9*) were also higher than the control group. It should be noted that GFAP transcription was reduced in the striatum of GFAP-Cre^ER^:Rbp-J^fl/fl^ mice relative to control Rbp-J^fl/fl^ after the ischemia. These results suggest that astrocytes transformation may occur in the absence of Rbp-J and contribute to the Rbp-J deletion-promoted neurogenesis in ischemic condition.

In summary, we successfully established a focal striatal ischemic model in adult mice via PT, and found that astrocytes and microglia increased in early post-ischemic stage, followed by a 1-week late-onset of DCX expression in the striatum. Then we examined a certain number of transcription factors and signaling pathways that have been reported to be involved in the striatal morphogenesis. Moreover, we provided the change of gene expression profile in the striatum of astrocyte-specific Rbp-J KO mice. Our data presented in this study may help to clarify potential mechanisms underlying brain ischemia-evoked neurogenesis. Adult neurogenesis in the hippocampus is highly activated in enriched environments, and it is of interest to investigate if a similar scenario is present in ischemia-induced transient neurogenesis in order to develop new therapeutic strategies for repairing brain functions caused by brain injury.

## Author Contributions

Z-ML and R-JZ contributed to the whole animal studies, the interpretation of the data, analysis of the data. X-SZ contributed to the interpretation of the data, analysis of the data and drafting the manuscript. YH and N-NS participated in the design of animal experiment, and the analysis of the data. J-YC contributed to the breeding and generation of the mice. Y-QD and C-JS designed and conceptualized the study. Z-ML and Y-QD wrote the manuscript. All authors prepared and approved the article for submission.

## Conflict of Interest Statement

The authors declare that the research was conducted in the absence of any commercial or financial relationships that could be construed as a potential conflict of interest.
