# Supplementary material for: Expression of DCX and Transcription Factor Profiling in Photothrombosis-Induced Focal Ischemia in Mice
Source: Front Cell Neurosci. 2018 Nov 22;12:455. doi: 10.3389/fncel.2018.00455 (PMC6262056; doi:10.3389/fncel.2018.00455)
Supplement: Supplementary file 1 [file Table_1.docx]

***Supplementary Material***

**Expression of DCX and Transcription Factor Profiling in Photothrombosis-induced Focal Ischemia in Mice**

**Zhu-Man Lv^1,2,6^, Rong-Jian Zhao^3,6^, Xiao-Song Zhi^4^, Ying Huang^2^, Jia-Yin Chen^2^, Ning-Ning Song^2^, Chang-Jun Su^3^*, Yu-Qiang Ding^1,2,5^***

***** **Correspondence**

Chang-Jun Su (changjunsu@163.com) or Yu-Qiang Ding (dingyuqiang@vip.163.com)

Supplemental Table1. The primer list for qRT-PCR

| Gene | Forward(5’→3’) | Reverse(5’→3’) |
| --- | --- | --- |
| Nkx2.2 | GCGGAGAAAGCATTTCAAAACC | GGACAAGCACCAAGCCAAAG |
| Nkx6.1 | AAACACACCAGACCCACGTT | GGTTCTGGAACCAGACCTTGA |
| Nkx6.2 | CGAGAGCCAAGTGAAGGTGT | TGTGCTTTTTGAGAAGCCGC |
| Foxp2  Tlx  SP8  SP9  Sfrp2  Er81  Gfap  DCX  Ascl1  Rbp-J  Neurod1  Adora2a  P2ry1  Grik3  Hes1  Hes5  Shh | TGAAACCGGGAAGTTTGCTCT  GCACAACCAATAGCCACCTG  AGGGCGGCCAGATCTAAGTA  TTTTGCCGGCGGGAGC  AGAGGAAGCTCCCAAGGTGT  TTTCTCTCGGCTCATCAGGAC  GCGAAGAAAACCGCATCACC  GAGTGGGGCTTTCGAGTGAT  GACTTTGGAAGCAGGATGGCA  GCCTGTTGTGACAGGGAAGTT  AACCTTTTAACAACAGGAAGTGG  CGCGGCCGGAGGTATC  TCAGAAGGAGACTGTCCCGA  CTGTGCTGGAGGAGCCTTTT  CAACACGACACCGGACAAAC  GAGAAAAACCGACTGCGGAA  TTTGGAAAGAGGCGGCACCC | CTGGAGAGCCTGCTGTTGTT  AAATGCGGCTTGTTGATCCG  CGAACCTTTCCTGTGCTTCC  CGTGGCTCTTCCCCAAGT  TTGAGCCACAGCACGGATTT  GAGGCCATGAAAAGCCAAACTT  TGGCAGGGCTCCATTTTCAA  GGTGGAACCACAGCAACTTTT  ACCCCTGTTTGCTGAGAACAT  GATACACACAAGGAGGAGGGC  GAGACACTCATCTGTCCAGC  CAGGGAGAGCTTCCCAAAGG  GCAGGTTCAAAGCAACCATGT  GTACTTGCCGTCTTCCACCA  GGAATGCCGGGAGCTATCTT  GCGAAGGCTTTGCTGTGTTT  TGCACCTCTGAGTCATCAGCC |
| β catenin | GTCAGTGCAGGAGGCCG | GGCCATGTCCAACTCCATCA |

Supplemental Table2. Antibody information

| Antibody | Dilution | Company |
| --- | --- | --- |
| Rabbit anti-GFAP | 1:1000 | DAKO |
| Ginea pig anti-DCX | 1:2000 | Millipore |
| Rat anti-BrdU | 1:1500 | Accurate chemical |
| Biotinylated horse anti-rabbit | 1:500 | Vector Laboratories |
| Biotinylated horse anti-ginea pig | 1:500 | Vector Laboratories |
| Donkey anti Rat-488 | 1:500 | Invitrogen |
| Cy3-conjugated reptavidin | 1:1000 | Sigma |
